# Case Report: Contrast-enhanced US and CT imaging features of nodule-in-nodule hepatocellular carcinoma in a dog

**DOI:** 10.3389/fvets.2026.1779996

**Published:** 2026-04-30

**Authors:** Mihyun Choi, Yongsun Kim, Kyuseok Choi, Jihun Won, Namsoon Lee

**Affiliations:** 1 BON Animal Medical Center, Suwon-si, Republic of Korea; 2 Section of Medical Imaging, Veterinary Medical Center, Chungbuk National University, Cheongju-si, Republic of Korea

**Keywords:** biopsy, dog, hepatocellular carcinoma, imaging, nodule-in-nodule

## Abstract

A spayed female Maltese dog weighing 3 kg found to have a hepatic nodule during a routine health examination. B-mode ultrasonography revealed a focal, round, heterogeneous mass located in the left lateral liver lobe, characterized by a target-like appearance with a hyperechoic center surrounded by a hypoechoic peripheral region. On contrast-enhanced ultrasonography, the lesion exhibited a central defect during the Kupffer phase. Multiphasic computed tomography (CT) demonstrated marked diffuse enhancement during the arterial phase, followed by heterogeneous washout in the portal phase. Delayed-phase CT images further revealed a hyperattenuating central nodule within a hypoattenuating peripheral component. To our knowledge, this is the first report describing the B-mode, ultrasonographic, contrast-enhanced ultrasonographic, and multiphasic CT features of nodule-in-nodule hepatocellular carcinoma in a dog, closely resembling imaging characteristics reported in human medicine.

## 1 Introduction

Hepatocellular carcinoma (HCC) is the most common primary hepatic neoplasm in dogs (1). Generally, canine HCC presents as the massive form, which is characterized by a large tumor involving a single liver lobe (2). On B-mode ultrasonography, HCCs are usually single and show variable echogenicity and echotextures, ranging from cystic to solid and from hyperechoic to hypoechoic or heterogenous, compared with the surrounding normal hepatic parenchyma (3). In contrast-enhanced ultrasonography (CEUS) using Sonazoid^®^, malignant hepatic lesions have been reported to demonstrate an enhancement defect during the liver-specific Kupffer phase (4). On computed tomography (CT), HCCs are commonly large and may contain areas of cystic degeneration or necrosis, with or without capsule formation. These tumors generally appear hypoattenuating during the portal and delayed phases compared with the surrounding liver parenchyma (5). In human medicine, the nodule-in-nodule (NIN) appearance is a well-recognized morphological imaging pattern that reflects the multistep process of hepatocarcinogenesis. This finding typically represents the dedifferentiation of HCC, whereby a less differentiated and more aggressive carcinoma develops within a pre-existing well-differentiated HCC or dysplastic nodule (6, 7). As diagnostic imaging plays a pivotal role in the non-invasive diagnosis of typical HCC, precise imaging characterization is equally critical for identifying the NIN pattern, which has important implications for predicting tumor malignancy (7). To the author's knowledge, the imaging characteristics of a hepatic mass exhibiting a mixed contrast enhancement pattern consistent with NIN appearance have not previously been reported in dogs. Herein, we describe the B-mode ultrasonographic, CEUS, and multiphasic CT features of a canine hepatocellular carcinoma exhibiting a NIN appearance.

## 2 Case description

A 7-year-old, spayed female Maltese dog, weighing 3 kg was found to have a hepatic nodule during a routine health examination. Physical examination revealed unremarkable findings, including body temperature, blood pressure, respiratory rate, capillary refill time and pulse rate. Complete blood count (CBC) results were within normal limits. Serum biochemical analysis revealed significantly elevated liver enzyme activities: ALT was 159 U/L [reference range (RR), 17–78 U/L], while ALP exceeded the upper limit of the analyzer's detection range (>3,500 U/L; RR, 47–254 U/L). Due to the marked elevation, the precise ALP value could not be determined within the standard laboratory parameters.

**Figure 1:**
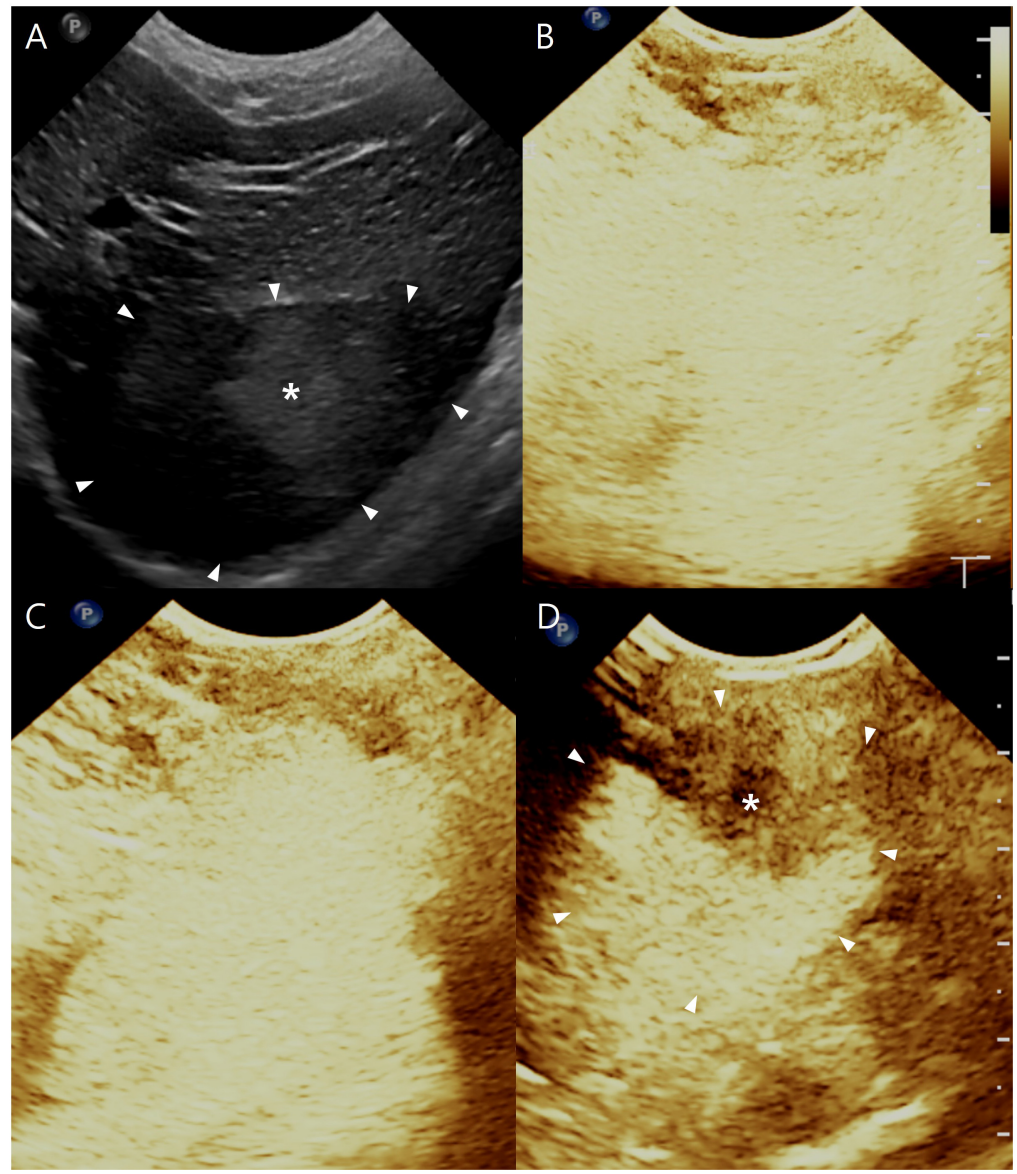
**(A)** shows a B-mode ultrasound image with a large hypoechoic area (white arrowheads) witn an inner hyperechoic liver lesion (asterisk). (**B**, arterial phase, 15 s) and (**C**, portal phase, 52 s) after administration of the microbubble contrast agent, show no remarkable findings compared with the surrounding liver parenchyma. (**D**, Kupffer phase) shows decreased enhancement of the inner nodule (white arrowheads) compared with the outer nodule (asterisk).

Abdominal and thoracic radiographic examination (1417WGC, Rayence Co., Ltd., Hwaseong-si, South Korea) revealed no remarkable findings. Abdominal ultrasound was performed using 4–18 MHz linear transducers (EPIQ 7 ELITE, Philips Healthcare, Amsterdam, The Netherlands). A focal, round, heterogeneous hepatic mass measuring 26 × 30 mm was identified arising from the left lateral liver lobe (Figure 1A). The mass showed target-like appearance, characterized by a smaller hyperechoic inner nodule surrounded by a larger hypoechoic outer nodule. The remaining hepatic parenchyma and hepatic lymph nodes appeared normal. No additional abnormalities were observed within the peritoneal cavity. Based on the ultrasonographic findings, differential diagnoses included nodular hyperplasia, hepatocellular adenoma, hepatocellular carcinoma, and less likely, other primary hepatic malignant tumors. To obtain further diagnostic information, CEUS was performed with a low mechanical index (0.2–0.3 MI). The gain was adjusted to the minimum level with which the liver parenchyma was slightly visible without contrast enhancement, and a single focal zone was positioned at the deepest region of interest. Sonazoid^®^ (Daiichi-Sankyo Co., Ltd., Tokyo, Japan), consisting of perfluorobutane within a hydrogenated egg phosphatidylserine shell, was administered intravenously at a dose of 0.015 ml/kg via the cephalic vein as a bolus injection, followed by a 3 ml saline flush containing 10% heparin. Post-contrast images were recorded as cine-loops for 1 min. Subsequently, whole-liver scanning was conducted in the same manner as conventional ultrasonography. Three contrast-enhanced phases (arterial, portal and Kupffer) were evaluated. During both the arterial and portal phase, the inner and outer nodules all showed contrast enhancement patterns similar to those of normal liver parenchyma. However, during the Kupffer phase, the inner nodule exhibited decreased enhancement compared to the normal liver, while the outer nodule demonstrated enhancement comparable to that of normal liver parenchyma (Figures 1B–D). Multiphasic helical CT was subsequently performed to further evaluate the hepatic mass.

**Figure 2:**
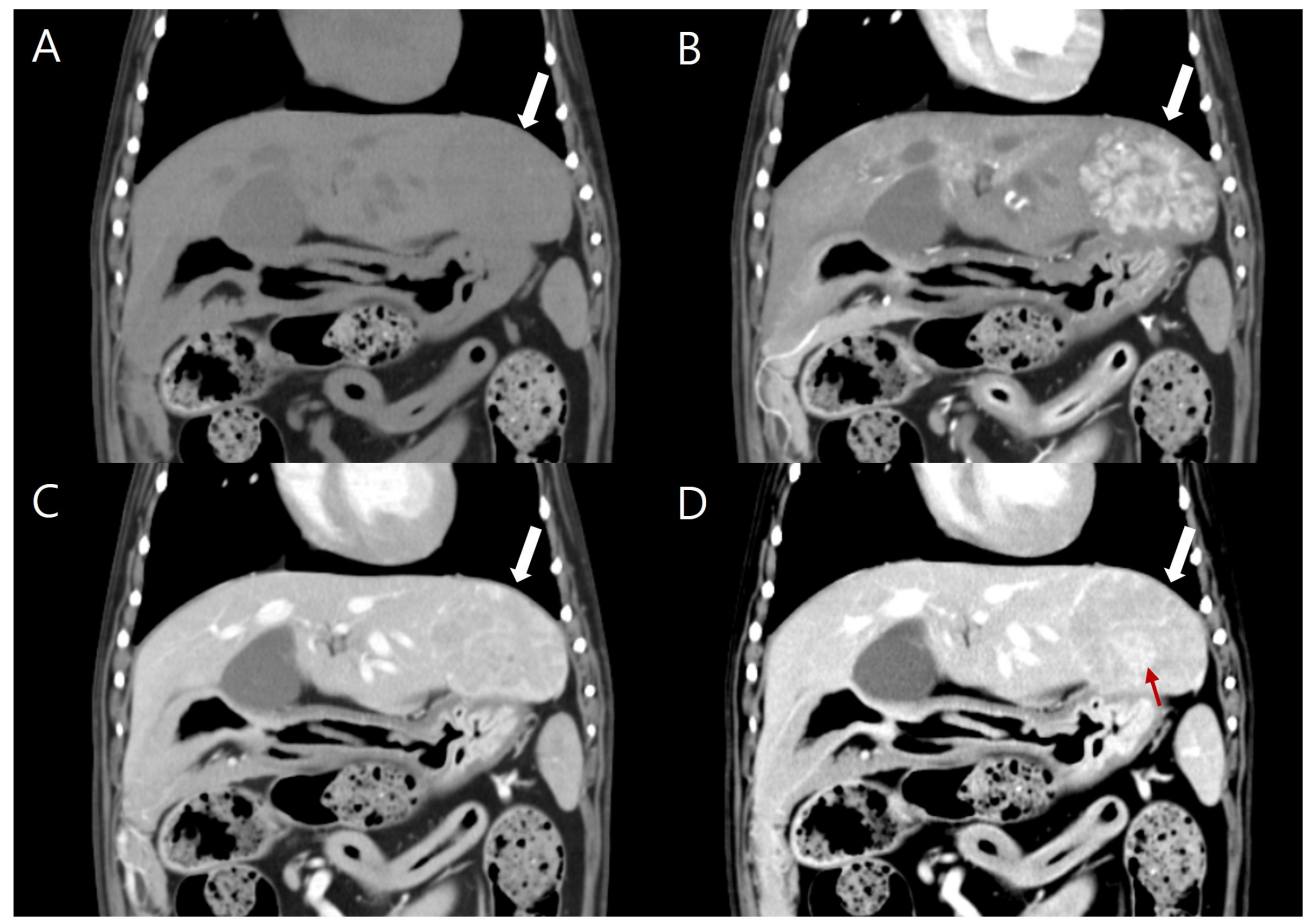
Pre-contrast **(A)** and post-contrast dorsal reformatted CT images obtained during the arterial **(B)**, portal **(C)** and delayed **(D)** phases using a soft tissue algorithm (Window level: 50 HU; window width: 400 HU). **(A)** Pre-contrast image shows an isoattenuating mass (white arrow), measuring 26 × 35 mm, in the left lateral liver lobe. **(B)** Arterial phase image shows early-wash in, diffuse enhancement and **(C)** portal phase shows early-wash out of the mass (white arrow). **(D)** The delayed phase shows a hyperattenuating inner nodule (8 × 10 mm, red arrow) and a hypoattenuating outer nodule (white arrow).

CT was performed using a 64-slice scanner (Aquillion64™, Canon Medical Systems, Tochigi, Japan). Anesthesia was induced with propofol (6 mg/kg, IV) and maintained with isoflurane. The CT acquisition parameters were as follows: slice thickness of 0.5 mm, tube voltage of 120 kVp, tube current of 150 mAs, a 512 × 512 matrix, and a rotation time of 0.75 s. For contrast-enhanced CT, iohexol (Omnipaque™ 300, GE Healthcare, Chicago, IL, USA) was administered intravenously at a dose of 700 mgI/kg using a power injector (Dual Shot GX-7, Nemoto Kyorindo co., Ltd., Tokyo, Japan) at an injection rate of 2.5 ml/s. A bolus-tracking protocol was applied for arterial-phase acquisition in a cranial- to caudal direction and was triggered when the attenuation of the aorta immediately caudal to the porta hepatis reached 250 Hounsfield units (HU), thereby, demonstrating the arterial phase. According to the standard procedure, the portal phase was acquired in a cranial to caudal direction 40 s after contrast administration. The delayed phase was acquired after 120 s. Images were reconstructed into 2.0 mm thickness transverse sequences with additional sagittal, dorsal, and oblique reformatted images generated using soft tissue and lung algorithms. On pre-contrast images, a large, homogenous, isoattenuating mass measuring 31 × 26 × 35 mm (height × width × length) was identified within the left lateral hepatic lobe (Figure 2A). The mass became distinct with diffuse enhancement in the arterial phase (Figure 2B), and followed by heterogeneous hypoattenuation relative to surrounding liver parenchyma in the portal phase (Figure 2C). In the delayed phase, the lesion exhibited a distinct nodule-in-nodule configuration, characterized by a hyperattenuating inner nodule and a hypoattenuating outer nodule (Figure 2D). The mean (SD) attenuation values of the inner nodule were 140.55 (30.30), 154.13 (10.43), and 157.75 (8.46) HU during the arterial, portal, and delayed phases, respectively. The corresponding values for the outer nodule were 177.04 (25.86), 160.69 (15.39), and 127.88 (8.25) HU. In comparison, the normal liver parenchyma measured 83.96 (9.62) HU, 169.61 (17.25) HU, and 136.48 (8.81) HU in the respective phases. Based on these values, the outer nodule demonstrated relative washout by the portal and delayed phases. In contrast, while the inner nodule's absolute attenuation increased progressively, it exhibited relative wash-out during the portal phase and became hyperattenuating in the delayed phase. Due to the larger size of the mass, the normal portal and hepatic veins of the left lateral lobe were mildly compressed. No additional abnormal lesions were detected in the remaining liver lobes, adjacent lymph node, or in the abdomen and thorax, and no metastases were suspected. Based on imaging findings, the differential diagnoses included primary hepatic tumors such as HCC and hepatocellular adenoma (HCA), but the inner nodular region, was suspected to be more malignant.

**Figure 3:**
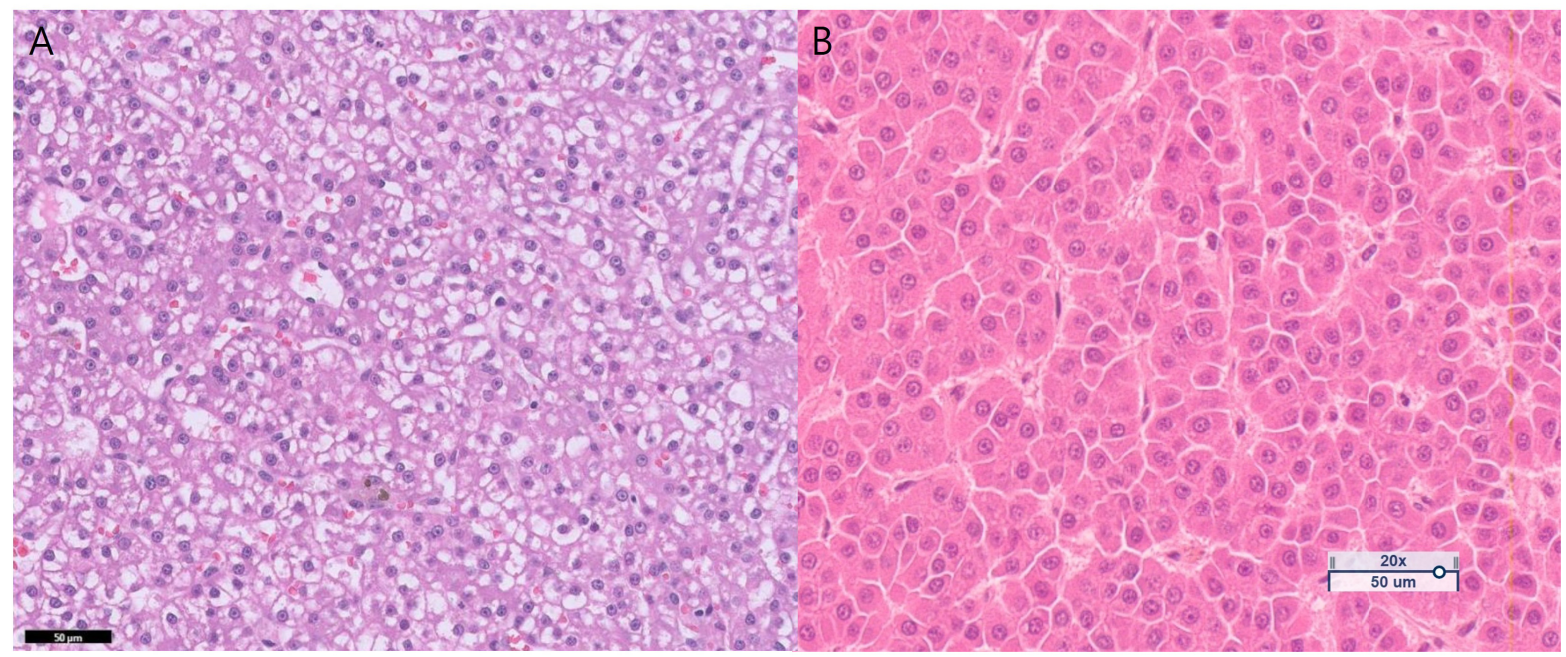
Histopathologic findings after HE staining of samples obtained by laparascopic biopsy **(A)** and liver lobectomy **(B)**. **(A)** Laparascopic sample confirmed a well-differentiated hepatocellular neoplasm accompanied by vacuolar hepatopathy. **(B)** Liver lobectomy sample confirms the diagnosis of hepatocellular carcinoma.

After CT scanning, a laparoscopic hepatic parenchymal biopsy was performed (Stryker 1288 HD, Stryker, Chicago, IL, USA). Anesthesia was induced with propofol (6 mg/kg, IV), and maintained using isoflurane in oxygen. The dog was continuously and appropriately monitored throughout the procedure. A two-port laparoscopic approach via the left upper quadrant was used. The patient was placed in right lateral recumbency, and a 6-mm subumbilical camera port was positioned in the left flank area, followed by placement of a second 6-mm instrument port in proximity. A 5-mm, 30-degree laparoscope was inserted, and before obtaining any biopsy sample, the diaphragmatic and visceral surfaces of all liver lobes were systemically examined for macroscopic lesions. Upon macroscopic evaluation, the mass in the left lateral liver lobe appeared diffusely pink and was associated with small numerous vessels. A 5-mm cup biopsy instrument was used to obtain two tissue samples from the dorsal part of left lateral liver lobe. The tissues from the hepatic parenchyma were fixed in 10% neutral-buffered formalin and submitted for histological evaluation at a laboratory (IDEXX Laboratories Inc., Westbrook, ME, USA). Histological examination (Figure 3A) showed a densely cellular, non-encaphulated, variably well-demarcated, multilobular mass with composed of polygonal cells, demonstrating marked hepatocytic differentiation and arranged in irregular trabeculae. Mild anisocytosis and anisokaryosis. The neoplastic cells exhibited distinct cytoplasm borders with abundant granular cytoplasm that was diffusely expanded by eosinophilic vacuoles. The biopsy samples confirmed well-differentiated hepatocellular neoplasm, with no mitotic figures observed per 10 high-powered fields. These findings raised suspicion for HCA accompanied by vacuolar hepatopathy; however, HCC could not be definitively excluded, and continued close monitoring was recommended. However, given that previous imaging results indicated more malignant features within the inner nodular region, a left lateral hepatic lobectomy was performed instead of close monitoring and submitted for histological evaluation at a laboratory (KVL Co Ltd., Seongnam, South Korea). Histological examination of the lobectomy sample (Figure 3B) showed polygonal hepatocyte-like cells, proliferating in a solid pattern, or forming trabeculae of variable thickness, and in some areas, exhibiting, a pseudograndular pattern. The tumor cells possessed abundant eosinophilic or vacuolated cytoplasm. Mild to moderate anisonucleosis was observed, and mitotic figures ranged from 0 to 1 per high-power field. No tumor emboli were identified in the examined sections. A definitive diagnosis of HCC with a complete tumor margin was established.

**Figure 4:**
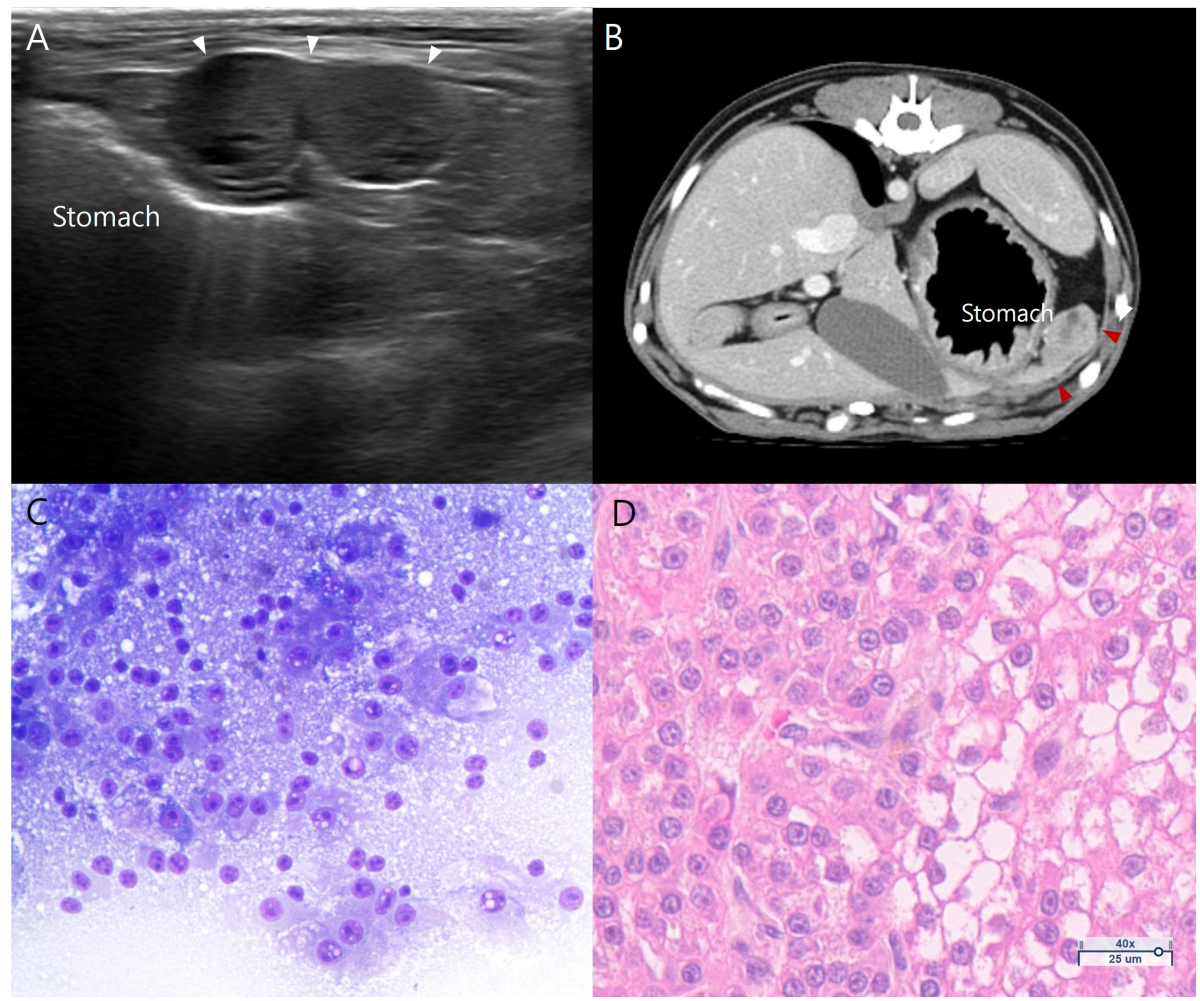
Peritoneal nodule (arrowheads) identified on **(A)** B-mode ultrasound, **(B)** Post-contrast axial CT images obtained during the portal phase, **(C)** fine-needle aspiration and **(D)** histopathologic images. **(A)** A bilobed shape, heterogenous, hypoechoic mass (arrowheads) is located between the stomach and spleen. **(B)** A heterogeneously enhancing soft-tissue nodule measuring, 9 × 10 × 16 mm is identified in the left upper abdomen near the previous surgical site (arrowheads). **(C)** Cytology examination suggests well-differentiated hepatocellular carcinoma (H&E, × 400). **(D)** Histopathologic examination, confirms metastatic hepatocellular carcinoma.

Fifteen months after the initial presentation, a follow-up ultrasonographic examination identified a peritoneal nodule of unknown origin, measuring 8 × 22 mm, located between the splenic tail and the stomach (Figure 4A). The nodule exhibited a bilobed shape with hypoechoic and heterogeneous echotexture. The remaining of the hepatic parenchyma and hepatic lymph node appeared normal. No additional abnormalities were observed within the peritoneal cavity on ultrasonography. A CT examination was performed with additional anatomical information. A soft-tissue-attenuating nodule measuring 9 × 10 × 16 mm was identified (Figure 4B) in the left upper abdomen, near the left medial hepatic lobe and splenic tail region. The lesion showed heterogeneous contrast enhancement. No additional abnormal lesions were detected within the thorax or abdomen, and no suspicion of metastasis was raised aside from this nodule. An ultrasound-guided fine-needle aspiration using 23-gauge needle was performed and the smears were stained with Diff-Quik (Sigma-Aldrich, St Louis, MO, USA). Cytologic examination (Figure 4C) revealed high cellularity, characterized by numerous round to polygonal hepatocyte-like cells. The nuclei were round and exhibited mild to moderate anisokaryosis, with coarse chromatin and occasional one to multiple prominent nucleoli. When interpreted in conjunction with the dog's prior diagnosis, these findings were consistent with metastatic HCC. After discussing treatment options with the owners, the nodule was surgically resected and submitted for histological evaluation at a laboratory (KVL Co Ltd., Seongnam, South Korea). Histopathological examination (Figure 4D) showed numerous irregularly sized hepatocytes like neoplastic cells diffusely distributed throughout the mass and arranged in thick trabeculae. A subset of neoplastic cells exhibited mild pleomorphism and karymegaly with irregular shape. A final diagnosis of peritoneal metastasis originating from the previously diagnosed HCC was confirmed.

## 3 Discussion

In human medicine, HCC develops through a multistep process, often in the context of chronic liver disease. This process referred to as hepatocarcinogenesis in cirrhotic livers, typically sequentially from regenerative nodules to dysplastic nodules (adenomatous hyperplasia), then to early HCC (subfocus of HCC), and finally to early advanced HCC (7). The distinctive NIN appearance observed in imaging studies (7, 8) represents the early advanced stage, in which a core of advanced HCC is pathologically surrounded by a rim of less advanced or early HCC. On CT scans, NIN lesions are characterized by a hypervascular, expansive inner nodule surrounded by an isovascular or hypovascular outer nodule during the arterial phase (8). Pathologically, the inner nodule is considered more aggressive than the surrounding outer nodule, which often contains borderline or malignant foci (9). The reported incidence of NIN appearances in early HCC with advanced foci detected on early-phase dynamic CT is approximately 33% (8, 10). The relatively low detection rate of NIN lesions is attributed to the rapid growth of the inner nodule. Several studies have reported that inner malignant foci have a volume doubling time ranging from 36 days to 9.5 weeks (8). When the inner malignant focus expands rapidly, the lesion may eventually lose its NIN pattern and transition to a massive HCC form. Considering that the most hepatic tumors in veterinary medicine are also discovered at a massive stage, the lack of reported NIN HCC in dogs is likely due to the limited use of advanced imaging when hepatic masses are still small, as identified in this patient.

The NIN pattern observed in our case exhibited distinct features compared with those typically reported in human medicine. In humans, the NIN lesions are characterized by hypervascular inner nodules surrounded by iso- or hypovascular outer nodules during the arterial phase on CT (8). Similarly, Sonazoid^®^-enhanced US demonstrates, an inner hypervascular spot followed by a Kupffer phase defect within a hypo- or isointense outer nodule (11). In contrast, the canine case presented here showed a progressive increase in attenuation across the arterial, portal, and delayed phases on CT. Although this pattern differs from the typical “washout” observed in many human hepatocellular carcinomas, some malignant lesions can demonstrate prolonged contrast retention due to specialized tumor vascularity or a prominent fibrous stroma (12). Crucially, the diagnostic interpretation was primarily guided by the Sonazoid^®^-enhanced US findings, where a distinct uptake defect in the Kupffer phase was observed within the inner nodule. In veterinary medicine, the absence of uptake during the Kupffer phase is a highly sensitive indicator of a lack of functional reticuloendothelial system (RES) cells (4). This finding provided definitive evidence for the malignant transformation of the inner focus, distinguishing it from the surrounding benign-appearing parenchyma despite the atypical CT enhancement.

Our case demonstrated a perceived discrepancy between the multiphasic CT findings and the CEUS enhancement patterns, which likely reflects the distinct pharmacokinetic properties of the respective contrast agents. While CT revealed early arterial enhancement and relative washout of the outer component, CEUS showed iso-enhancement during the early vascular phases. CT contrast media, such as Iohexol^®^ extravasate into the interstitial space (13), potentially highlighting differences in interstitial volume or capillary permeability between the inner and outer nodules. In contrast, Sonazoid^®^ microbubbles remain strictly intravascular during the vascular phase (14). This allows CEUS to provide a more specific assessment of the microvascular and cellular environment—most notably, the loss of functional Kupffer cells. This specific finding characterizes malignant transformation in dogs (4), providing a more definitive diagnostic basis than the interstitial-phase-dependent findings of CT.

The NIN pattern may be explained by two potential mechanisms. First the pathogenesis could resemble human “nodule-in-nodule” HCC, where foci of advanced HCC develop within pre-existing early HCC. However, this hypothesis is limited due to the inherent differences in HCC mechanisms between humans and dogs. Second, and more plausibly the lesion may represent malignant transformation of a pre-existing HCA. In human clinical practice, HCAs are classified into four principal subtypes based on the 2006 criteria (15): HNF-1α-inactivated HCA (H-HCA), inflammatory HCA (I-HCA), β-catenin-activated HCA (B-HCA), and unclassified HCA (U-HCA). The risk of complications, such as internal bleeding and malignant transformation, is closely associated with specific subtype. Notably, the β-catenin-activated subtype (B-HCA) carries a high risk, with approximately 46% of cases progressing to HCC or an intermediate borderline lesion, whereas the HNF-1α-inactivated subtype (H-HCA) demonstrate the lowest incidence of both hemorrhage and malignant transformation (16). Although the distinct subtypes are not yet recognized in veterinary medicine, reports exist regarding the needle-tract seeding of hepatocellular tumors and malignant transformation of HCA into HCC (17). In our case, the outer nodule exhibited features consistent with benign tissues compared to the malignant inner nodule (HCC), strongly suggesting a mechanism similar to the malignant transformation observed in high-risk human HCA subtypes.

The pathogenesis has significant clinical implications. First, histologic interpretation of laparoscopic biopsy requires caution, the sample may only represent the benign outer layer (HCA) and fail to capture the inner malignancy. Second, in evaluating the abdominal nodule detected 15 months post-surgery, differentiating between metastasis originating from the inner HCC and delayed malignant transformation of seeded cells remains challenging. However, given that the primary mass was resected with clear margins, it is plausible that benign HCA cells seeded at the laparoscopic entry site subsequently underwent malignant transformation, mirroring the pathology of the primary tumor.

In human clinical practice, magnetic resonance imaging (MRI) is the preferred modality for HCC evaluation, largely due to its ability to provide detailed information when hepatobiliary-specific contrast agents like Gadoxetic acid (Gd-EOB-DTPA), are used. This technique, known as EOB-MRI, serves as an imaging biomarker for HCC, allowing for the accurate visualization of both hemodynamic and cellular metabolic changes within the inner and outer components of NIN HCCs (18). The inner nodule typically exhibits characteristics associated with increased malignancy, including T2-hyperintensity, restricted diffusion, and a rapid arterial phase wash-in followed by wash-out enhancement. In contrast, the surrounding outer nodule generally appears less aggressive, demonstrating iso- or hypointensity on T2-weighted images, minimal or no restricted diffusion, and hypovascularity during the arterial phase (19). Human studies using Gd-EOB-DTPA-enhanced MRI have identified several lesions that resemble the NIN pattern (20, 21). One such lesion is the doughnut-like nodule (sometimes referred to as multiacinar cirrhotic nodules). These differ from NIN lesions because they exhibit absent arterial phase enhancement, a phenomenon potentially related to non-uniform impairment of intrahepatic portal blood flow (20). Another mimic is the enhancing variant of focal nodular hyperplasia, in which central hypointensity on EOB-MRI is attributed to both reduced enhancement within the central scar tissue and in the surrounding hepatocytes (21).

This case illustrates the diagnostic challenges associated with hepatic masses exhibiting a NIN pattern. While imaging revealed a heterogeneous structure, the initial laparoscopic biopsy captured only benign tissue. This discrepancy suggests that intratumoral heterogeneity may lead to sampling bias, particularly in complex lesions where malignant foci are nested within benign components. Although a precise topographic correlation between the biopsy site and the subsequent histopathology was not definitively established, our findings highlight the potential for superficial sampling to underrepresent the internal complexity of such masses.

A primary limitation of this report is the lack of a precise topographic correlation between the histopathology and imaging findings. The distinction between the outer and inner nodules was inferred based on the sampling location, as the histological transition zone could not be definitively visualized with the precision often reported in human literature. Furthermore, the diagnostic significance of the NIN pattern remains poorly characterized in canine hepatocarcinogenesis. While this pattern is a well-recognized marker for malignancy in humans, its biological behavior in veterinary patients has not been validated through large-scale studies. Consequently, our interpretation of these imaging findings as representing a specific evolutionary stage of the tumor remains speculative and necessitates further clinicopathological validation in a larger canine cohort.

In conclusion, while canine HCC typically presents as a massive tumor characterized by internal necrosis and hemorrhage, this case indicates that an NIN pattern, a recognized feature of multistep hepatocarcinogenesis in humans, may also occur in dogs. Recognition of this pattern may be clinically relevant when evaluating hepatic masses. In lesions exhibiting such structural heterogeneity, where a malignant inner focus may be nested within a benign outer component, caution is warranted during tissue sampling. A limited biopsy targeting only the superficial aspect of the lesion may not fully represent the internal malignancy, potentially leading to an underestimation of the disease grade. Therefore, when an NIN pattern is identified via imaging, a more comprehensive sampling strategy may be considered to facilitate a more representative diagnosis.

## Data Availability

The original contributions presented in the study are included in the article/supplementary material, further inquiries can be directed to the corresponding author.
